# WeChat dual-group (mis-)use: a qualitative exploration of Chinese undergraduate students’ use of mobile social media applications and its pedagogical implications

**DOI:** 10.3389/fpsyg.2025.1686513

**Published:** 2025-12-10

**Authors:** Yuanxing Tan, Ran Ren

**Affiliations:** 1 College of Foreign Languages and Cultures, Chengdu University of Technology, Chengdu, China; 2 Department of Sociology, Chengdu University of Technology, Chengdu, China

**Keywords:** collaborative learning, English translation education, second language learning, discipline construction, social media applications, programme development

## Abstract

**Introduction:**

Although WeChat is widely adopted in Chinese higher-education English-translation programmes, little is known about how students actually use its group-chat function for collaborative learning or what problems arise. This study explores the dual-group phenomenon—parallel instructor including inclusive and student-only WeChat groups, to uncover both the merits and the challenges of this informal digital ecology.

**Methods:**

An interpretivist, qualitative design was employed. Twenty-eight undergraduates from a Chinese technology-focused public university were recruited through course announcements, email and flyers. Semi-structured, face-to-face interviews were conducted, transcribed, translated and analysed inductively using thematic analysis.

**Results:**

All participants reported maintaining two distinct WeChat groups per course: (1) an authoritative, instructor-inclusive channel for official notices and verified resources; and (2) an informal, student-only space for peer troubleshooting, social support and rapid resource sharing. Benefits included clear role differentiation, emotional safety for “naïve” questions, and accelerated knowledge co-construction. Downsides were information overload, off-topic chatter, fragmented communication, unequal participation dominated by vocal peers, and limited instructor oversight. Half of the informants observed instances of academic misconduct (e.g., sharing quiz screenshots or copying translations). Students also noted blurred professional boundaries, with some expecting 24/7 instructor availability.

**Discussion:**

The dual-group arrangement constitutes a layered communication ecology that simultaneously enhances and undermines collaborative learning. While it balances authority with autonomy, its unregulated nature risks cognitive overload, inequity and integrity breaches. Examples of pedagogical recommendations include: (a) explicit norms for each group, (b) weekly pinned summaries to reduce noise, (c) peer moderators to ensure equitable voice, (d) assessment designs that deter cheating, and (e) co-created digital-communication codes to clarify collaboration–plagiarism boundaries. Institutionally, embedding digital professionalism into curriculum standards and establishing discipline-level hubs for digital-translation pedagogy can better achieve university learning goals.

## 1 Introduction

Over the past two decades, social media platforms have become integral to how people communicate and collaborate, extending beyond social interaction to support personal, professional, and educational activities. Their affordances—such as instant messaging, multimedia sharing, and real-time feedback—make them attractive tools for collaborative learning in higher education (Tess, 2013; Perez et al., 2023). In second language education, such platforms foster interaction-driven learning and enable flexible, multimodal engagement across locations and time zones (Akbari et al., 2015; Lee, 2022). Yet these benefits also raise questions about how openness and immediacy can be balanced with purposeful communication and academic integrity (Kukulska-Hulme and Viberg, 2018).

In China, WeChat has become a particularly influential platform for enabling such collaboration in second language learning (Huang, 2019; Wang et al., 2021; Wu and Ding, 2017). Combining instant messaging, multimedia sharing, group management tools, and integration with other digital services, WeChat has become deeply embedded in the daily routines of students and educators. Prior research has documented its benefits, ranging from improving fluency and pronunciation in spoken English and writing performance to enriching cultural awareness (e.g., Dai et al., 2018; Li and Liontas, 2023; Huang, 2019; Shi et al., 2017; Sun and Asmawi, 2023; Wang, 2017; Wang and Jiang, 2021; Wu and Ding, 2017; Wu and Miller, 2021; Yan, 2019). Although these studies have demonstrated the pedagogical benefits of social media platforms in second language education, less attention has been given to the both the benefit and pedagogical challenges that accompany their increasing adoption in collaborative learning in English translation programmes. In particular, we know little about the downsides of use of mobile social media applications in such context. The lack of critical investigation into potential issues is significant because the rapid institutional adoption of WeChat and similar platforms in higher education seem to outpace the development of pedagogical understandings, posing potential challenges for language teaching and productive participation.

To address these gaps, this study offers a qualitative exploration of Chinese undergraduate students’ use and misuse of WeChat group chats in the context of English translation education. Drawing on in-depth interviews with 28 students at a public university, it investigates how these students use and experience WeChat group chats while completing their learning tasks and coursework, and what the merits and downsides of this use are. Specifically, it examines the emergence of a “dual-group” arrangement in which instructor-inclusive groups operate alongside student-only groups and considers the implications of this layered communication ecology for collaborative learning.

By situating these findings within the framework of collaborative learning, the study not only provides empirically grounded insights into how ubiquitous social media applications are used and managed, especially in terms of its merits and downsides. Such understandings can address the aforementioned gap in the literature and enrich our understandings about the use of group chat functions of these platforms. In addition, the paper discusses how the use of group chat functions and the associated collaborative learning can be managed to balance immediacy and accessibility. It advances practical understanding of how instructors can engage with student-generated digital spaces in the face of the challenges revealed by this study, such as fragmented communication, diminished instructor oversight, and blurred boundaries. The critical and practice-oriented implications underscore that by establishing communication norms, integrating peer-generated insights into formal learning, and embedding digital professionalism into curriculum design, instructors and institutions can transform WeChat from a casual coordination tool into a structured pedagogical resource that strengthens both collaborative learning and programme/disciplinary development of English translation teaching.

## 2 Digital-technology-assisted collaborative learning in second language education

Collaborative learning, grounded in socio-cultural (Vygotsky, 1978) and social interdependence theory (Johnson and Johnson, 1991, 2009), emphasises interaction, mutual support, and co-construction of knowledge. In second language education, it promotes communicative competence through authentic peer dialogue and shared task completion (Zhang, 2010; Zhang et al., 2013). These principles are further elaborated within socio-cultural theory (Vygotsky, 1978, 1986; Zhang et al., 2013), which posits that learning is socially mediated and occurs within the zone of proximal development, where language is shared and internalised through mediation. This process enables learners to reach higher levels of language development through scaffolding (Vygotsky, 1978, p. 86). Meanwhile, social interdependence theory proposes that the structure of learning goals determines the nature of interaction among learners. Positive interdependence emerges when individuals perceive that their success is linked to that of others, fostering promotive interaction, mutual support, and shared responsibility in language learning (Johnson and Johnson, 2009).

Now, collaborative learning can be facilitated and enhanced through the integration of digital tools, which can create further interaction, resource sharing, and joint problem-solving among learners (see Golonka et al., 2014; Hasumi and Chiu, 2024; Kreijns et al., 2003; Su and Zou, 2022, Zhang and Zou, 2020, for review). These tools, ranging from learning management systems to social media platforms, offer both synchronous and asynchronous modes of communication, enabling students to work together across temporal and spatial boundaries. A prominent subset of digital-technology-assisted collaborative learning is the form of learning assisted by mobile messaging platforms (Power, 2018; Zhang, 2019). Social media applications, in particular, have become increasingly significant in supporting collaborative learning, especially in contexts where informal and semi-formal communication plays a central role (see Barrot, 2022; John and Yunus, 2021; Liu, 2024 for review). Through text, voice notes, images, video, and file sharing, these platforms create multi-modal channels for collaborative exchange, enabling peers to provide feedback, share resources, and coordinate joint tasks (Chen, 2022; Huang, 2019; Wang et al., 2021; Wu and Ding, 2017). They support both synchronous interaction, allowing for rapid clarification, brainstorming, and decision-making, as well as asynchronous engagement that gives learners time to reflect before responding and accommodating different schedules (Wang et al., 2016).

Recent studies have also demonstrated the significant impacts of digital collaborative learning environments, such as platform-mediated learning and AI-human interaction, on students’ English learning. For instance, Ebadijalal and Moradkhani (2025) investigated the effects of computer-assisted collaborative writing (CW), collaborative prewriting (CPW), and individual writing (IW) on the performance and motivation of Iranian EFL learners. Their study found that CW and CPW significantly improved learners’ writing performance and motivation compared to IW. Similarly, Wang (2025) explored the impact of an online collaborative argumentation environment (CAE) on EFL students’ argumentative writing skills, showing that students in the CAE group demonstrated significant improvements in incorporating substantial grounds and effective rebuttals in their essays. Pratama et al. (2025) found that the integration of AI-assisted tools significantly improved students’ abilities to construct well-structured arguments and use critical thinking skills. Chen and Huang (2025) identified various factors influencing learner engagement, including group roles, perception of Automated Writing Evaluation feedback, writing ability, and prior writing knowledge. Pratama et al. (2025) also emphasized that the human-AI collaborative writing strategy fostered positive student perceptions and experiences, particularly regarding the ease of the writing process, writing enjoyment, and writing satisfaction.

In this study, collaborative learning is deemed not only a key purpose and outcome of using mobile social media applications, but also a conceptual frame for this qualitative exploration. In other words, by qualitatively investigating how Chinese undergraduate students use and misuse WeChat groups in the process of completing their daily learning tasks and coursework, this paper aims to understand not just the patterns of use, but also the extent to which WeChat group chats can facilitate or undermine the construction of positive collaborative learning in English translation education. As such, this study can offer a case to further reflect on the pedagogical challenges and directions of change in the face of widely application of WeChat in second language education, as we have little knowledge about how WeChat group chats can facilitate or challenge digital collaborative learning.

## 3 The use of WeChat in learning English as a second language

In recent years, the integration of digital communication platforms into higher education has become increasingly prevalent, particularly in non-Western contexts where mainstream global platforms may be less dominant. In mainland China, WeChat has emerged as a ubiquitous tool not only for social interaction but also for academic coordination and pedagogical practices. As a multi-functional mobile application combining instant messaging, file sharing, voice messaging, group management features and even AI-assisted searching, WeChat has become the *de facto* infrastructure for informal and semi-formal communication across many domains of Chinese university life, including second language education at the university level (e.g., Dai et al., 2018; Li and Liontas, 2023; Huang, 2019; Shi et al., 2017; Sun and Asmawi, 2023; Wang, 2017; Wang and Jiang, 2021; Wu and Ding, 2017; Wu and Miller, 2021; Yan, 2019).

In terms of learning English as a second language, a growing body of studies suggests a positive role of WeChat use in shaping mobile learning, which in turn increases native cultural awareness (Wu and Miller, 2021), strengthens learner autonomy and attitude (Dai et al., 2018), improves pronunciation and fluency of spoken English (Wang, 2017; Wang and Crosthwaite, 2021), enhances vivid phrasal idiom learning (Li and Liontas, 2023) and writing performance (Sun and Asmawi, 2023; Yan, 2019). For instance, Wu and Miller (2021) have explored how WeChat can be used to raise native cultural awareness among Chinese EFL students through an informal mobile learning community, in which the Community of Practice concept positively influenced students’ native cultural awareness, with participants becoming more willing to engage in discussions and share their cultural knowledge. In studying the function of WeChat moments, Wang and Jiang (2021) indicate that the WeChat Moments group showed significantly better writing performance compared to the control group, particularly in task response, grammatical range and accuracy.

Despite these fruitful findings on the prevalence of WeChat use and its positive outcomes, little is known about how undergraduate students in English majors use WeChat to facilitate collaborative learning in the process of completing individual and group tasks and assessments and what the consequences of their behaviours and interactions on WeChat groups are for collaborative learning and digital pedagogy. In this sense, it is necessary to explore the real-world experiences and agency of university students in respect of their (mis-)use of WeChat functions, particularly its group chats. Therefore, by utilising the case of undergraduate students from English translation programmes, this paper offers an account of their experiences in terms of the merits and downsides of group chat use as well as the concerns these practices raise for instructors.

## 4 Research context of this study

Among Chinese higher education institutions, WeChat group chats are widely used to organise class activities, disseminate course-related materials, facilitate student collaboration, and coordinate both individual and group-based tasks (e.g., Huang, 2019; Qi and Wang, 2018; Wang et al., 2021; Wu and Ding, 2017). Instructors often use WeChat groups as extensions of the classroom, either supplementing or replacing more formal learning management systems. These digital environments provide students with readily accessible channels for peer-to-peer engagement, clarification of assignment expectations, exchange of resources, and submission of work. Particularly in language and translation programmes, which involve solitary text production, vocal exchanges, and interactive collaborative projects, group-chat platforms serve as dynamic spaces of academic interaction and cooperation.

At the public university where this study was conducted, a leading Chinese public institution with a strong emphasis on technology and science, WeChat is not merely common but has become normalised as a default mode of class communication and coordination. Both faculty and students rely on WeChat group chats to distribute notices, clarify course requirements, assign tasks, and facilitate ongoing interactions beyond the physical classroom. While an Official Administrative System is available, students and instructors overwhelmingly favour WeChat for its immediacy, flexibility, and familiarity. Each course typically establishes a dedicated WeChat group at the beginning of the course module, with the instructor either actively participating or delegating announcements to student representatives. This organisational norm reinforces the WeChat’s ubiquity and embeds it deeply in the everyday academic routines of undergraduate students. As such, the case setting offers a representative and meaningful site for investigating the cognitive and social dimensions of digitally mediated academic communication in second language learning.

For students in English literature and translation programmes, the use of WeChat is further contextualised by the smaller cohorts of classmates, where annual intakes of students in these programmes are around 70 to 80, and there are usually around 30 students in each course class of English translation. Such a small size of cohorts creates a suitable context for digital collaborative learning and pedagogy, in which WeChat could facilitate a digital space conducive to effective interaction between instructors and students.

## 5 Methods

This study aims to investigate undergraduate use of group-chat applications in the field of advanced English translation learning from the perspective of university students. It is situated within an interpretivist epistemology, which assumes that knowledge of social phenomena is co-constructed through participants’ meanings and researchers’ interpretive engagement. The study adopts a constructionist ontology and a sociocultural theoretical perspective, viewing learning as a socially mediated process shaped by contextual interactions and shared practices. This orientation underpins our use of qualitative, inductive methods to explore how students experience and make sense of their digital learning environments. The interpretivist stance aligns closely with the study’s theoretical grounding in collaborative and sociocultural learning theories, which emphasise meaning-making, interdependence, and mediation through social interaction (Vygotsky, 1978; Johnson and Johnson, 1991, 2009). Accordingly, the methodological choice of thematic analysis was guided by the goal of identifying patterned meanings within participants’ accounts that reflect their lived experiences of WeChat-mediated collaboration. While, to date, the application of group-chat applications has been discussed in the literature of second language learning chiefly in connection with quantitative examination of performance results (e.g., Wang, 2017; Wang and Crosthwaite, 2021; Wu and Miller, 2021; Sun and Asmawi, 2023), limited knowledge is known about the experiences of learners in the process of completing individual and group learning tasks and relevant implications for students and instructors. Therefore, an exploratory qualitative design was adopted (Creswell and Poth, 2018).

This study sampled the students via course announcements, departmental email notices, and classroom flyers, in which the introductory and consent documents for this project were included. The first author employed this method of purposive sampling to answer our research questions on the basis of recruitment for undergraduate students (aged 18 and above) who are enrolled in the full-time programmes of English translation and have regularly used WeChat groups to engage in daily learning and assessments. As a result, 28 students were recruited and interviewed by the first author, at which point thematic saturation was reached (Creswell and Poth, 2018). In this sample of students, 12 were male and 16 female; 5 were first-year students, 10 s-year, and 8 final-year (see Table 1 for further detail).

**Table 1:** Informant profile.

ID	Informant	Gender	Year of study	Recruitment source
1	Chloe	F	Year 1	Email
2	Harry	M	Year 1	Email
3	Leah	F	Year 1	Flyer
4	Ryan	M	Year 1	Referral
5	Yara	F	Year 1	Email
6	Alex	F	Year 2	Referral
7	George	M	Year 2	Referral
8	Ivy	F	Year 2	Email
9	Jack	M	Year 2	Referral
10	Maya	F	Year 2	Referral
11	Olivia	F	Year 2	Flyer
12	Tia	F	Year 2	Referral
13	Zach	M	Year 2	Email
14	Christiana	F	Year 2	Flyer
15	Brian	M	Year 2	Referral
16	Emma	F	Year 3	Course
17	Karen	F	Year 3	Course
18	Noah	M	Year 3	Course
19	Quinn	F	Year 3	Email
20	Sophia	F	Year 3	Course
21	Victor	M	Year 3	Referral
22	Wendy	F	Year 3	Course
23	Daniel	M	Year 3	Course
24	Fiona	F	Year 4 (final)	Flyer
25	Paul	M	Year 4 (final)	Email
26	Uma	F	Year 4 (final)	Referral
27	Xavier	M	Year 4 (final)	Referral
28	John	M	Year 4 (final)	Referral

Interview data of interest were collected through in-depth, tape-recorded, semi-structured face-to-face interviews conducted throughout an academic year (September 2024–June 2025). A core interview guide (see details of this question list in the Appendix) focusing on the prevalence of WeChat usage and concomitant experiences and perceptions was constructed to ensure the thematic coherence and direction of discussion, while flexible probes allowed participants to elaborate on issues they deemed salient (Creswell and Poth, 2018). This approach was essential given the limited nature of prior scholarship: it would have been neither feasible nor desirable to prescribe an exhaustive question list *ex ante*. Each interview lasted at least 60 min, involving detailed accounts of WeChat usage, perceived advantages and drawbacks, and broader reflections on individual experiences.

Data analysis was carried out by both authors. To analyse our interview data, we assigned an English pseudonym to each informant to ensure anonymity, transcribed the Chinese interviews and then translated relevant excerpts into English. Subsequently, we conducted inductive coding to develop code categories and identify thematic patterns in the use of WeChat functions for completing course assessments (Braun and Clarke, 2006). As a result of a back-and-forth process of data collection and analysis, we induced three major themes, including the merits of use, the downsides of use, and concerns for instructors. To ensure analytic rigour and consistency, both authors independently coded the first six interview transcripts and compared their initial code sets. Coding discrepancies were discussed in joint meetings until a shared understanding of code boundaries and meanings was reached, leading to the refinement of the coding framework. The remaining transcripts were then coded using this agreed framework. Periodic cross-checks were conducted to ensure alignment, and any interpretive differences were resolved through iterative discussion and consensus rather than statistical calculation, in line with qualitative reliability principles (Braun and Clarke, 2006). Thematic saturation was assessed continuously during data collection and analysis. Following Braun and Clarke’s (2006) inductive approach, we monitored the emergence of new codes and themes after each set of three to four interviews. No new thematic categories or substantive variations in existing ones appeared after the 26th interview, at which point coding stability was confirmed through the final two interviews (27th and 28th). These last interviews served to verify rather than expand the thematic structure, indicating that thematic saturation had been achieved. The interview quotes in the following sections illustrate the common viewpoints of informants.

To enhance the trustworthiness of the analysis, we adopted several validation strategies. Although formal member checking with participants was not undertaken, both authors engaged in ongoing peer debriefing throughout the analytic process to review coding decisions, challenge interpretations, and refine thematic definitions. In addition, analytic notes and code development records were maintained as an informal audit trail, ensuring transparency in how themes evolved from raw data to the final structure (Creswell and Poth, 2018).

Both authors are lecturers in higher education with extensive experience teaching in Chinese universities. The first author, trained in translation studies, conducted all interviews and approached the analysis with a focus on learners’ communicative practices and digital engagement. The second author, a sociologist of education, contributed to the interpretation of data through a broader lens of social interaction and institutional norms. We acknowledge that our dual insider perspectives, as instructors familiar with WeChat-mediated pedagogy, may have sensitised us to certain pedagogical and interactional dynamics while potentially overlooking taken-for-granted practices. To mitigate this bias, we adopted a reflexive and iterative approach throughout the analysis, engaging in critical peer debriefing to challenge assumptions and ensure that interpretations remained grounded in participants’ accounts.

## 6 Findings

A consistent pattern across interviews was the maintenance of two separate WeChat group chats for each course. The first group chat, created and managed exclusively by students, served as an informal space for daily learning exchanges and peer-to-peer communication. Here, students discussed translation strategies, clarified assignment requirements, shared drafts or glossaries, and occasionally coordinated study schedules. This student-only group was also a site for social bonding, where participants exchanged jokes, encouragement, or personal updates alongside academic talk. Such informality allowed for candid conversations without the perceived pressure of instructor oversight, fostering what one informant described as a “safe zone” for asking “naive” questions or admitting confusion.

The second group chat was a more formal channel jointly established by students and instructors for occasional, targeted communication. This space was primarily used for official announcements, resource dissemination, and clarifications issued by the teaching team. Students described the instructor-inclusive group as “authoritative” and “instruction-driven,” noting that messages here carried more weight and demanded prompt attention. While interaction in this space was less frequent, its function was widely recognised as critical for maintaining course organisation and ensuring that key information reached all participants. The coexistence of these two parallel communication spaces reflects students’ ability to strategically compartmentalise media environments by separating collaborative peer learning from hierarchical, formalised exchanges, thereby utilising both the social and instructional affordances of WeChat.

**Figure 1:**
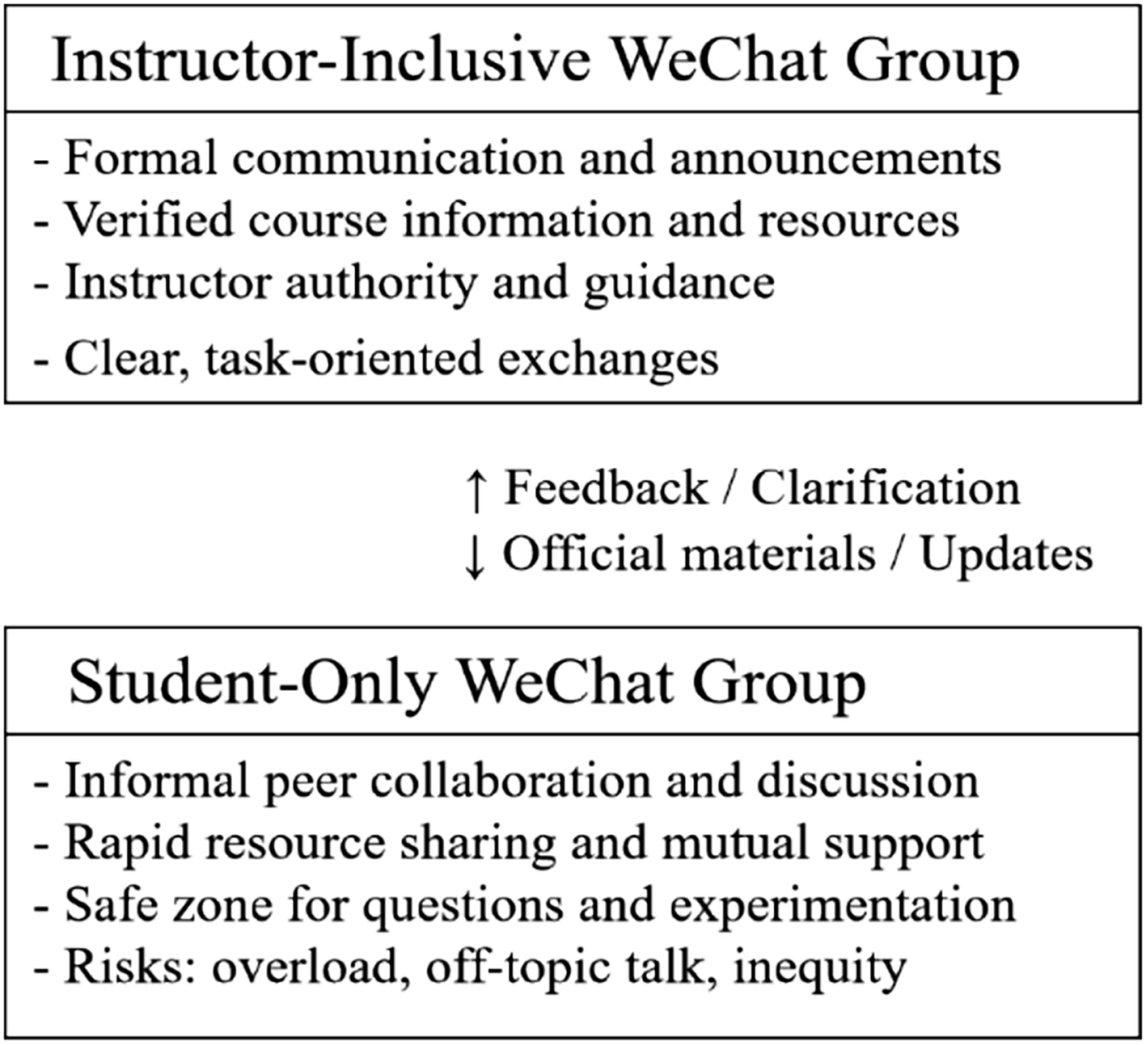
The layered communication ecology of WeChat-mediated collaborative learning.

The analysis induces these dynamics as a layered communication ecology in which instructor-inclusive and student-only groups operate as interrelated yet distinct spaces for digital collaboration. To illustrate this structure more clearly, Figure 1 provides a schematic representation of the two-layer system and their respective affordances. This layered communication ecology highlights how Chinese undergraduates strategically navigate between two overlapping yet functionally differentiated communication layers. The upper (instructor-inclusive) layer ensures accuracy, structure, and pedagogical authority, while the lower (student-only) layer fosters autonomy, emotional safety, and immediacy. The bidirectional arrows indicate information and relational flows between layers, where peer discussions often precede formal clarification, and instructor feedback sometimes informs peer reinterpretation. The model demonstrates how both layers together sustain a dynamic, dual-channel ecosystem of collaborative learning.

### 6.1 Merits of use

There are a few major merits of this use of WeChat group chats in facilitating different dynamics of group collaborative learning. First, many informants highlighted how maintaining two separate WeChat groups allowed them to leverage the platform’s full range of functions for different purposes. For instance, Maya explained:

“If I want to confirm a detail, like the exact deadline for our assignments, I’ll go straight to the teacher’s group. Everything there is formal and clearly from the teacher. But if I want to discuss how to translate a tricky idiom or share an example sentence, I’ll post in our own group. That’s where the discussion is more relaxed and we can go back and forth without worrying about drawing teacher’s attention”. (Maya, F, Year 2)

Maya’s account illustrates students’ ability to strategically navigate two digital spaces: one oriented toward authoritative, instructor-led communication, and another toward collaborative, peer-driven learning. This structural separation allowed participants to utilise both the instructional affordances (accuracy, clarity, official information) and social affordances (mutual support, shared problem-solving) of WeChat. The dual-group structure described by participants aligns closely with established principles of collaborative learning. The student-only WeChat groups play a key role in shaping peer learning communities in which members co-constructed knowledge, offered mutual feedback, and engaged in joint problem-solving without the hierarchical constraints of teacher-led spaces. In light of our data, informal exchanges, such as “*brainstorming tricky idioms*” (Maya, F, Year 2), or “*cultural nuances between Chinese notions and English ones*” (Leah, F, Year 1), show how these digital interactions operate as “the zone of proximal development” for collaborative meaning-making and absorbing (Johnson and Johnson, 2009; Vygotsky, 1978). Such exchanges are not merely social; they are embedded in cognitive work that advances individual and collective understanding of translation tasks.

Second, according to these students, such dual-group model also serves as a way to keep messages purposeful and to maintain a clear boundary between informal and formal interactions for communication clarity and safe peer learning environment. As Victor and Karen explained:

“When the teacher is in the group, everyone writes shorter messages and stay low key. We only post if it’s about assignments, deadlines, or important class issues. (But in our own group) we’ll sometimes send voice notes to explain things, share jokes, or even talk about internship opportunities. Having both groups keeps the teacher’s group free from noise, and our group free from feeling too serious”. (Victor, M, Year 3)

“Sometimes I don’t want to post in the main class’s group because it feels like everyone will see, and I don’t want to ask something that seems too basic. In our own group, I can say, ‘I don’t get what the assignment means by cultural equivalence and mediation, and someone will just explain or send a screenshot of their notes”. (Karen, F, Year 3)

Victor’s reflection represents the views of most students, underscoring how role differentiation between instructor and peer spaces contributes to communication clarity. On the other hand, Karen’s experience points to the importance of low-pressure environments for learner engagement in formal concepts and codified knowledge in a flexibly coordinated way. By compartmentalising channels, students reduce the risk of burying critical academic information in casual exchanges, while still sustaining a separate space where interpersonal ties can flourish. Importantly, this differentiation between the two spaces addresses a frequent challenge of balancing information density with social presence. By keeping the instructor-led group focused on high-priority academic messages, participants ensured that critical announcements were not lost in the noise of informal chatter. Meanwhile, the peer-only space sustained the interpersonal bonds and low-pressure digital space known to enhance engagement and participation in cooperative learning and digital pedagogy environments (Johnson and Johnson, 1991; Kukulska-Hulme and Viberg, 2018).

Third, informants valued the instructor-inclusive group for its efficiency in distributing accurate, official materials, while more informal resource sharing and dissemination was championed in student-only groups. Noah and Sophia described their use of two Wechat groups:

“Our translation assignments had a lot of small corrections to the source text. The course instructor, like your course, posted the corrected version in the group instead of emailing it. That way, everyone got it at the same time and we could start working on the same version immediately”. (Noah, M, Year 3)

“In our own group, we sometimes share resources faster than the teacher’s group. For example, when someone found a good bilingual glossary for the exam, they just dropped the link and the file right away. Within minutes, everyone was downloading it”. (Sophia, F, Year 3)

Noah’s viewpoint emphasises how the group format levels the playing field in access to information, ensuring simultaneous receipt and minimising inequities in preparation time. Sophia’s comment demonstrates that while official instructor-led groups provide authoritative materials, the student-only group can also function as a rapid, peer-driven resource network, often extending the range of materials available and responding flexibly to learners’ immediate needs. These accounts indicate that efficient resource dissemination and sharing in the dual-group system depends on both the official instructor-led channel, which ensures accuracy and consistency, and the student-only channel, which provides speed, responsiveness, and supplementary materials. This dual mechanism reflects how learners actively manage multiple digital spaces to enhance access to and use of information for completing course assignments and assessments. In doing so, they have effectively created a layered digital learning ecosystem: one layer focused on precision and authority, the other on social interaction and mutual support, which maximised the pedagogical potential of a ubiquitous use of WeChat.

Beyond this functional distinction, several participants described these dual groups as serving complementary pedagogical and psychological purposes. As Yara (F, Year 1) explains, “Having a separate group for us means we can double-check things before bothering the teacher; it’s like a rehearsal space where we build confidence.” This notion of a “rehearsal” environment demonstrates how peer-only spaces not only facilitated resource exchange but also offered emotional safety for experimenting with linguistic and conceptual ideas before formal submission. Similarly, Leah (F, Year 1) notes that such exchanges helped “make translation feel less intimidating,” showing how informal collaboration supports engagement and self-efficacy. These insights further reaffirm the positive alignment between peer support and collaborative meaning-making.

### 6.2 Downsides of use

Without exception, there were also notable downsides to the use of WeChat groups, which appear rather pronounced in student-only groups where sustained collaborative learning may not always take a productive form. First, while participants valued the speed and accessibility of WeChat communication, several noted that the sheer volume of messages, particularly in the student-only group, could be overwhelming and overloaded. As Wendy and Brian suggested:

“Some days, there are more than a hundred message notifications, so I have to mute it. (I found) Half of them are about the assignment, but the other half are random things, like someone sharing a meme. If you don’t check it constantly, you miss the important messages in between. It takes time to scroll back and find what you actually need”. (Wendy, F, Year 3)

“We had to submit our translation draft by Friday. On Wednesday, the group was so noisy that I didn’t see much useful information, but only complaints and questions”. (Brian, M, Year 2)

The comments from Wendy and Brian show how high-frequency posting can dilute the visibility of critical information, creating extra cognitive and organisational work for participants. Even though the platform affords immediacy, the absence of message filtering tools means that participants must manually sift through long message chains to locate relevant content. The issue of information overload reflects a common pitfall in digital collaborative learning: when message volume is high and content is mixed in relevance, the cognitive load for participants increases, diverting attention from the substantive academic task (Kreijns et al., 2003; Kukulska-Hulme and Viberg, 2018).

Second, the above issue further reflects a deeper challenge in student-only groups: fragmentation of communication and going off-topic. The existence of two separate groups also sometimes led to reduced discipline of engagement; and confusion of information sharing and the informal group could easily drift off-topic. As Ivy and Harry offered relevant comments:

“Sometimes someone will share a useful document in our group, at least I think it’s useful, but it never appears in the teacher’s group. Then I’m not sure if the teacher knew about that or assessed (the material)”. (Ivy, F, Year 2)

“Two days before our presentation, people were posting about SNS posts in the middle of discussing our slides. It’s distracting, and sometimes you just give up scrolling because it feels like a waste of time”. (Harry, M, Year 1)

Their comments show that fragmentation risked creating parallel knowledge streams, with little further assessment from teaching authority to verify the usefulness of new learning resources and materials. Furthermore, off-topic chatter can reduce the efficiency of collaborative spaces, a common tension in digitally mediated peer groups where social and academic purposes overlap. Loss of coherence in communication and the tendency for discussions to drift off-topic echo concerns in the literature on online learning communities about maintaining coherence and discipline to sustain a sense of community of learning and a orderly space of learning with clear purposes (Kreijns et al., 2003; Kukulska-Hulme and Viberg, 2018). Without explicit strategies to centralise and reconcile information and manage group flows, participants must expend extra effort to piece together a complete picture.

Third, not all participants felt equally empowered to contribute. The emergence of dominant contributors can inadvertently silence others, who could even translate into subtle forms of exclusion. As Quinn vividly revealed her observation in her student-focused groups:

“There are a few classmates who always speak first and post the most. Sometimes they answer before you can even type, and it feels like your point isn’t needed anymore. After a while, I just stopped replying unless someone asked me directly. (…) If you’re not in the circle of people who talk the first or the most, sometimes your questions get ignored. I once asked about a translation example, and no one replied until one of that ‘main’ people commented”. (Quinn, F, Year 3)

Her comment shows that dominance of certain voices in group chats could lead to passive participation and reduced diversity of perspectives, and even a sense of exclusion or marginalisation of less connected participants. Such group dynamics could undermine the effectiveness of equitable knowledge exchange in online collaborative learning and prevent active engagement and contribution (Johnson and Johnson, 1991, 2009).

Several additional informants elaborated on the psychological and organisational strain caused by message overload. Fiona (F, Year 4) observed that “sometimes messages just keep coming and you lose track of what’s serious and what’s just gossip; it makes me anxious before deadlines.” Jack (M, Year 2) also commented that “if you miss a few hours, it’s like entering a flood, you do not know where to start.” While Fiona’s experience highlights the emotional cost of maintaining vigilance in a high-volume communication environment, Jack’s comment underscores the cognitive load created by fragmented threads. These patterns suggest that while WeChat’s immediacy enhances accessibility, it can simultaneously undermine focus and sustained collaboration — a tension central to understanding digital peer learning dynamics.

### 6.3 Concerns for instructors

Although this study only included the interviews with students, their responses raise some concerns for instructors teaching at the undergraduate level. WeChat can pose some real potential problems that may affect pedagogical effectiveness in English translation. Three issues that emerged from the interview discussions were loss of oversight in student-only groups, cheating in coursework and exams, and blurred boundaries of teacher-student interactions.

First, given that student-only groups often served as valuable peer learning spaces, instructors had no visibility into the accuracy or appropriateness of what was shared there, as also evidenced in earlier interview excerpts. As Christiana further showed that the absence of instructor oversight may allow unverified or even incorrect information to circulate, “*Some students share grammar explanations or background information of literature texts from random websites or AI-assisted searches, and I’m not sure they are correct. But because the teacher is not in the group, we cannot check immediately. And some students just use those sites to do your homework without verification*.” Daniel also offered an example of missed pedagogical opportunities:

“We had a long discussion about whether a sentence should be translated literally or with cultural adaptation. The teacher would probably have given us a better explanation, but she wasn’t in the group, so it just stayed between us”. (Daniel, M, Year 3)

His comment suggests that valuable peer discussions in student-only spaces may remain invisible to instructors, limiting their ability to guide learning or integrate these insights into formal teaching. These findings connect to a known issue of “hidden” or “shadow” learning spaces (Kreijns et al., 2003; Su and Zou, 2022; Zhang and Zou, 2020). While these private peer networks can foster candid exchanges and quick information-sharing, the lack of instructor involvement means that misconceptions may go uncorrected and high-value discussions may not be integrated into the formal learning process. The separation of digital spaces can reduce the instructor’s ability to provide scaffolding at moments when it might most effectively advance learning (Vygotsky, 1978, 1986; Zhang et al., 2013).

Second, the loss of oversight could also cause forms of cheating, including students sharing homework answers and cheating on translation exams. When asked about cheating, about half the informants reported seeing cheating behaviour on WeChat, and about half did not. Typically, more senior students and more heavy users were more likely to report that they had seen cheating behaviour on WeChat. For instance, Noah and Emma revealed the prevalence of this misuse:

“Before a vocabulary translation quiz, a few people in our group would send screenshots of the test questions as soon as they finished. Others who hadn’t started yet could see them and prepare the answers. It’s not everyone, but it happens, especially for online tests”. (Noah, M, Year 3)

“A few students posted their finished translation in the group, and others just copy parts of it. They might change a few words, but it’s still basically the same work. I think the teacher doesn’t know because it’s not so obvious”. (Emma, F, Year 3)

In this sense, these digital spaces may also blur the line between collaboration and academic misconduct, presenting a significant integrity challenge for instructors, particularly in low-proctored or remote assessment contexts. The affordances of instant, multimodal communication can be appropriated for purposes that undermine collaborative learning’s core principle of positive interdependence (Johnson and Johnson, 2009). The WeChat-based sharing of completed translations or exam answers reflect a form of collaborative dishonesty, where group solidarity and collaboration are misapplied to enable academic misconduct (McCabe et al., 2012).

Third, in mixed (student-instructor) groups, the casual tone and immediacy of messaging as well as direct access to instructors’ accounts sometimes led to a blurring boundary in instructor-student interactions. For example, some students expected that instructors would be constantly available and could instantly reply to their messages. As Paul observed, such expectation may undermine professional boundaries and increase instructor workload, especially in digitally informal teaching outside classroom schedule:

“Some classmates would message the teacher at midnight and expect a reply right away. If the teacher didn’t answer, they started wondering or asking each other if she’s read it yet. It’s likely that they forget teachers have their own life”. (Paul, M, Year 4)

Other informants also noted that the relaxed environment of WeChat groups could occasionally lead to unprofessional behaviours. As Chloe recounted that this sort of informality may erode the perceived authority of the instructor or the academic focus of the group:

“In the main (student-instructor) group, people sometimes send stickers or emojis that are funny, but (it) might be too casual for talking to a teacher. It can make the group feel less serious”. (Chloe, F, Year 1)

The issue of blurred boundaries of teacher-student interactions reflect a challenge of role definition and communication etiquette in technology-mediated environments, where establishing interaction norms and building interactional order to avoid “perpetual contact” expectations or unprofessional conducts is needed. Without an orderly interaction, the professional and pedagogical authority can be undermined seriously, thereby negatively affecting the construction of collaborative learning community.

Additional interview evidence also revealed students’ awareness that their informal discussions might hold pedagogical value unrecognised by instructors. As Olivia (F, Year 2) noted, “The lecturer does not always know how much we actually discuss outside class; if she saw it, maybe she’d guide us better, or gave us more specific and tailored advice”. This perception indicates that informal WeChat exchanges, while often pedagogically rich, remain peripheral to formal teaching evaluation. Such accounts reinforce the need for structured mechanisms to bridge informal and formal learning spaces, thereby maximising the pedagogical potential of students’ digital collaborations.

## 7 Conclusion and discussion

This study examines how undergraduate students in English translation programmes at a Chinese public university use WeChat group chats to organise coursework, collaborate on assignments, and sustain both academic and social communication. Drawing on in-depth qualitative interviews with 28 participants, the analysis revealed a dominant dual-group model: an instructor-inclusive group used for authoritative announcements and formal course coordination, and a student-only group serving as an informal space for peer learning, resource sharing, and social support. This division of digital space allowed students to utilise WeChat’s social and instructional affordances, creating a layered learning ecosystem that balanced accuracy and authority with collaboration and camaraderie. The arrangement supported efficient dissemination of resources, role differentiation, and the cultivation of a safe peer learning environment conducive to active engagement in translation learning.

This study makes two main contributions to research on social media-mediated collaborative learning. Empirically, it identifies and analyzes a dual-group communication structure in real-world teaching settings, which has been largely overlooked in prior literature (e.g., Dai et al., 2018; Li and Liontas, 2023; Huang, 2019; Shi et al., 2017; Sun and Asmawi, 2023; Wang, 2017; Wang and Jiang, 2021; Wu and Ding, 2017; Wu and Miller, 2021; Yan, 2019). This layered arrangement shows how students deliberately separate formal, authoritative exchanges from informal, peer-driven collaboration to balance accuracy, resource control, and social support. While grounded in English translation programmes at a Chinese public university, this model offers a transferable lens for examining digitally mediated collaboration in other disciplines and contexts among Chinese universities. Theoretically, the findings illuminate the gist of collaborative learning by demonstrating how learners actively reconfigure platform affordances to construct parallel communication spaces with distinct norms, roles, and functions (Dillenbourg, 1999; Johnson and Johnson, 1991, 2009). This “layered communication ecology” extends current understandings of human-social media interaction in educational settings and invites further comparative research across platforms, cultures, and subject areas.

Nevertheless, this study also has several limitations. The single-institution, discipline-specific sample limits the generalisability of the findings to other contexts. Reliance on self-reported accounts means some behaviours, particularly those involving academic misconduct, may have been under-reported or selectively framed. The absence of instructor perspectives also leaves unexplored how faculty manage and integrate such communication channels into their pedagogy and manage the associated workflows. Furthermore, we recognise that the findings are derived exclusively from student accounts, which inevitably reflect their perceptions and self-reported experiences. Without complementary data from instructors, certain phenomena — particularly instances of academic dishonesty — may be subject to perceptual bias or overstatement. Students may have interpreted peer activities through limited observation or assumption, while instructors might frame or evaluate these behaviours differently.

Future research could further advance this line of inquiry by incorporating instructors’ perspectives, which would provide complementary insights into how teaching staff perceive, manage, and respond to dual-group communication dynamics. Including digital communication trace data, such as anonymised message logs or interaction patterns, could enable the triangulation of self-reported experiences with behavioural evidence, thereby enriching the validity of findings. Moreover, adopting an elongated or longitudinal design would help assess whether students’ WeChat-mediated collaboration and communicative norms remain stable, evolve, or diminish over time. Such multi-source and time-extended approaches would not only strengthen empirical verification but also illuminate the sustainability and pedagogical consequences of digitally mediated collaborative learning practices.

## 8 Pedagogical implications

This study also reveals notable limitations and challenges in the normalised use of WeChat for facilitating collaborative learning in undergraduate English translation programmes. In student-only groups, information overload and off-topic chatter often obscured key updates, while dominant voices could marginalise quieter members and reduce active, equitable participation. The separation between groups sometimes led to fragmented communication, leaving valuable resources and discussions unseen by instructors or the wider cohort. From a teaching perspective, concerns included the loss of oversight, the facilitation of academic misconduct, and blurred professional boundaries in mixed groups, where the casual tone and expectations for instant replies added to instructors’ workloads. These patterns highlight the double-edged nature of ubiquitous digital platforms in university teaching—where benefits for collaborative learning are tempered by risks of inefficiency, inequity, and compromised academic integrity.

The pedagogical implications of these findings point to the need for deliberate structuring and management of seemingly separate digital communication spaces. Instructors can capitalise on the strengths of the dual-group system by clearly defining the purposes, norms, and expectations for each channel, ensuring that the instructor-inclusive group remains the authoritative source of verified information while encouraging student-only groups to function as productive spaces for discussion and resource sharing that enhance the learning of translation theories and techniques. Mechanisms should also be established to integrate valuable peer-generated content from informal spaces into the formal learning environment. Attention should be paid to managing cognitive load and information flow through periodic summaries, pinned messages, or topic-specific threads to prevent important updates from being buried in high-volume chats. Safeguarding academic integrity requires assessment designs that minimise opportunities for answer sharing, for instance, by conducting on-site exams, quizzes, or presentations, while simultaneously promoting authentic collaboration and open dialogue with students about the ethical use of digital tools. Finally, clear boundaries and professional etiquette for instructor–student interactions in WeChat groups are essential to preserve respect and balance workload. By addressing these areas, educators can more effectively harness the potential of WeChat as both a formal and informal learning tool, maintaining the benefits of immediacy, accessibility, and peer support while mitigating the risks associated with unregulated or poorly structured digital spaces.

Under this framework, instructors can adopt a range of practical strategies to manage the dual-group learning ecology more effectively. Implementing a weekly pinned summary in the instructor-inclusive group can consolidate key announcements, resources, and clarifications, thereby reducing message overload and ensuring that essential information remains easily retrievable. Appointing peer moderators within student-only groups can promote equitable participation, maintain topic relevance, and facilitate the summarisation of valuable discussions to be shared with instructors. Developing a digital communication and integrity code at the beginning of each semester, co-designed with students, can define appropriate online behaviour, clarify the distinction between collaboration and plagiarism, and foster a sense of shared responsibility for maintaining academic honesty. Instructors may also conduct periodic reflective check-ins or short feedback polls to gauge how students use these spaces, adjusting their guidance accordingly. As such, these measures can sustain the advantages of immediacy and autonomy in WeChat-mediated learning while strengthening transparency, accountability, and professional respect across both communication layers.

Beyond the classroom level, the findings also carry implications for discipline construction and programme development in English translation education. As digital technologies increasingly shape translation practice and pedagogy, the discipline must evolve toward a more integrated model that combines linguistic expertise, digital literacy, and intercultural communication competence. Incorporating WeChat-mediated collaboration into disciplinary development plans can enhance students’ readiness for digitally networked translation work, aligning university training with contemporary industry practices. From an institutional perspective, departments might consider establishing research and practice hubs dedicated to digital translation pedagogy to consolidate innovation, share cross-course resources, and produce evidence-based guidelines for the use of social media in translation learning. At the level of discipline construction, embedding digital professionalism and ethics into standards for curriculum evaluation and teacher development can further strengthen the quality of English translation programmes. Together, these initiatives position WeChat and similar digital communication tools not as peripheral aids but as integral components of the evolving disciplinary ecosystem of translation education.

## Data Availability

The datasets presented in this article are not readily available because the interview data is not allowed to share, due to the ethical restriction regulated by the Research Ethics Committee at the College of Foreign Languages and Cultures, Chengdu University of Technology. Requests to access the datasets should be directed to Yuanxing Tan, Windsor0125@126.com.
