# Supplementary material for: WeChat dual-group (mis-)use: a qualitative exploration of Chinese undergraduate students’ use of mobile social media applications and its pedagogical implications
Source: Front Psychol. 2025 Dec 10;16:1686513. doi: 10.3389/fpsyg.2025.1686513 (PMC12728073; doi:10.3389/fpsyg.2025.1686513)
Supplement: Supplementary file 1 [file Supplementary_file_1.docx]

Appendix. The Interview Guide

| **Topics** | **Opening Questions** | **Optional Probes** |
| --- | --- | --- |
| **Intensity of Use** | “Can you describe how often and in what ways you typically use WeChat for your coursework?” | • “Are there certain types of assignments or moments in the semester when WeChat is used more often?”  • “Do you use it more for group assignments, individual tasks, or both?”  • “Is this something you do daily, weekly, or only occasionally?” |
| **Motive of Use** | “What usually motivates you to use WeChat for class-related purposes?” | • “Is it more about convenience, habit, social pressure, or something else?”  • “Do you use it because your classmates expect you to be there?”  • “How do your instructors or course expectations influence your use?” |
| **Experience of Use** | “Can you recall a few specific experiences that stand out to you when using WeChat for your studies?” | • “Was there a moment when it helped you feel more connected or more confused?”  • “What roles do you usually play in group chats—are you active, passive, organising, observing?”  • “Has anything surprised you in the way information flows through these chats?” |
| **Reflection on Use** | “Looking back, how do you feel about using WeChat for learning? What does it add or take away?” | • “Do you think it helps or hinders your learning in English translation?”  • “What would you change about how WeChat is used for coursework, if anything?”  • “Are there moments when you’ve thought: I wish we had done this differently?” |
